# Bone Marrow Failure in Prostate Cancer: A Rare Case Initially Disguised as Thrombotic Thrombocytopenic Purpura

**DOI:** 10.7759/cureus.80781

**Published:** 2025-03-18

**Authors:** Sunil R Dommaraju, Michael Shodiya, Kathryn Henick

**Affiliations:** 1 Division of General Internal Medicine and Health Services Research, Department of Medicine, UCLA Ronald Reagan Medical Center, Los Angeles, USA; 2 Division of Hematology-Oncology, Department of Medicine, UCLA Ronald Reagan Medical Center, Los Angeles, USA; 3 Division of Hematology/Oncology, Department of Medicine, VA Greater Los Angeles Healthcare System, Los Angeles, USA

**Keywords:** bone marrow failure, incidental prostate cancer, prostate cancer (pca), rare cause of thrombocytopenia, ttp (thrombotic thrombocytopenic purpura)

## Abstract

Prostate cancer is the second most common cause of cancer-related death and the leading cause of new cancer cases in men in the United States. Bone trabeculae are the most common site of metastasis for prostate cancer, but bone marrow (BM) involvement is rare. Around six percent of patients with new metastatic castrate-sensitive prostate cancer (mCSPC) have biopsy-proven BM metastasis. We present a case of mCSPC with BM metastasis initially disguised as thrombotic thrombocytopenic purpura (TTP). Initial presentation revealed anemia and thrombocytopenia with a peripheral blood smear indicating thrombotic microangiopathy. Empiric treatment for TTP was initiated, but the patient showed no improvement. A BM biopsy performed on hospital day 5 for further work-up of the refractory cytopenias eventually identified metastatic prostate adenocarcinoma. The patient started androgen deprivation therapy, but attempts at follow-up care were unsuccessful. The patient passed away five months later. This case highlights the diagnostic complexity of cytopenias and contributes to our understanding of BM-metastatic prostate cancer. BM involvement in mCSPC complicates prognosis, and further research into its pathophysiology and targeted therapies is needed to improve patient outcomes.

## Introduction

Prostate cancer (PCa) is the second most common cause of cancer-related death in men and the leading cause of new cancer cases in men in the United States [1]. In 2025, an estimated 313,780 new cases of PCa will be diagnosed in the United States and 35,770 men will die from the disease [1]. PCa most commonly presents with urinary frequency and hesitancy, nocturia, dysuria, and in advanced disease, it presents with hematuria, erectile dysfunction, weight loss, and bone pain. However, atypical presentations have been reported [2], and one systematic review reports that patients with lower urinary tract symptoms are not at higher risk of developing PCa than those who were asymptomatic [3]. While there is an overall five-year relative survival rate of 97.5% based on data between 2014 and 2020 [4], the five-year survival drops to around 29.8% in metastatic disease [5]. The most common metastatic sites are bone (84%), distant lymph nodes (10.6%), liver (10.2%), and thorax (9.1%) [6]. Prognostic factors that correlate with poorer outcomes for patients with metastatic disease include poor performance status, shorter prostate-specific antigen (PSA) doubling time, higher Gleason score, pain at presentation, high tumor volume, and visceral metastasis [7].

Still, only up to 10% of patients have bone metastases at initial diagnosis. The presence of bone marrow (BM) metastasis in PCa is even rarer, as demonstrated by one study, which showed that of those with new metastatic castrate-sensitive prostate cancer (mCSPC), only 6% had biopsy-proven BM involvement at the time of diagnosis [8]. While BM metastasis in several solid tumors has been well-characterized and known to result in cytopenias due to marrow replacement and disruption of routine signaling pathways, the clinical significance and prognostication of BM involvement in PCa remains unclear. We present the case of a 91-year-old man who presented with chronic rib fractures, weight loss, and thrombotic microangiopathy (TMA). He was empirically managed for thrombotic thrombocytopenic purpura (TTP) but, after lack of improvement and normal ADAMTS13 (a disintegrin and metalloproteinase with thrombospondin type 1 motif, 13) level, was eventually diagnosed with mCSPC with BM involvement.

## Case presentation

A 91-year-old Caucasian male with a pertinent history of Graves’ disease status post (s/p) radioactive iodine therapy complicated by hypothyroidism on levothyroxine, muscle-invasive bladder cancer diagnosed 12 years ago s/p radiation therapy without evidence of recurrence, urge urinary incontinence and benign prostatic hyperplasia (BPH) with chronic indwelling Foley catheter, a four-year history of low-risk immunoglobin M (IgM) monoclonal gammopathy of undetermined significance (MGUS), and a recent (two years) diagnosis of ringed sideroblastic myelodysplastic syndrome (MDS) with single lineage dysplasia on surveillance was brought to the emergency department with altered mental status (AMS) and right-sided chest pain.

On initial evaluation, the patient was disoriented and incoherent. Vital signs were stable except for a temperature of 101.4°F. Physical examination was notable for tenderness along the right-sided ribs. Acute coronary syndrome was ruled out with a stable electrocardiogram and normal troponins. A bilateral three-view X-ray of the ribs revealed an angulation of the right lateral 10th rib representative of a fracture as well as multiple chronic left-sided rib fractures, a stable chronic right distal clavicle fracture, and a stable chronic compression fracture at the first lumbar vertebra. These were managed supportively with a multi-modal pain regimen. The patient also noted a 30-pound weight loss over an uncertain period.

Labs showed a marked hemoglobin (Hgb) and platelet drop from baseline seven months prior to presentation (Table 1). The rest of the complete blood count showed a normal white blood cell count with normal differential, an elevated mean corpuscular volume, elevated red cell distribution width, and elevated percent reticulocyte count. His coagulation studies were within normal limits. Remaining labs were notable for elevated lactate dehydrogenase (LDH) and low haptoglobin. Chemistry was pertinent for elevated aspartate aminotransferase and total bilirubin (with normal direct bilirubin). Direct Coombs test was negative. The peripheral blood smear showed normocytic red blood cells (RBCs) with anisocytosis, three+ (moderate) schistocytes, and teardrop forms. A urinalysis was concerning for active urinary tract infection. The patient was admitted to the general medicine service with AMS secondary to urinary tract infection and with TMA concerning for TTP.

**Table 1:** Serial laboratory values from the first 12 hours of hospital stay

Test	Baseline values (7 months prior)	Results (day 1 evening)	Results (day 2 morning)	Reference range
Hemoglobin	10.5	7.9	6.2	13.3-17.7 g/dL
White blood cell count	6.83	5.36	5.55	4.5-11.0 k/uL
Platelets	359	83	50	150-440 k/uL
Mean corpuscular volume	106.8	101.3	100.5	80-99 fL
Red cell distribution width	16.8	23.1	23.0	12-15%
Absolute reticulocyte count		0.05		0.025-0.090 M/uL
Percentage reticulocyte count		2.28		0.5-1.5%
Peripheral blood smear		2+ schistocytes	3+ schistocytes	None
Prothrombin time		17.6		12.0-13.8 seconds
Partial thromboplastin time		31.8		26.7-34.6 seconds
International normalized ratio		1.39		2.0-3.0 ratio
Lactate dehydrogenase		767	953	87-271 U/L
Haptoglobin		<8		43-212 mg/dL
Direct antiglobulin, polyspecific		Negative		Negative
Aspartate aminotransferase		377		13-35 U/L
Alanine transaminase		9	12	7-45 U/L
Alkaline phosphatase		50	39	33-94 U/L
Total bilirubin		1.2	1.5	0.2-1 mg/dL
Direct bilirubin			0.4	0-0.4 mg/dL

The hematology-oncology service was consulted, and they shared the concern for TTP or worsening MDS. ADAMTS13 activity level, serum protein electrophoresis (SPEP), quantitative immunoglobins (QIGs), serum free light chains, and serum viscosity were sent. Although “PLASMIC” score for TTP was only 4, serial labs over 12 hours showed worsening of thrombocytopenia, anemia, and indirect hyperbilirubinemia with rising LDH (Table 1), and thus the patient was started on 1-gram intravenous methylprednisolone and daily plasma exchange (PLEX). However, the patient continued to require a total of five units of packed red blood cells over the next few days. On day 5, BM biopsy was performed given the lack of improvement and to further evaluate suspected progression of MDS. On day 6, ADAMTS13 level resulted and was nearly normal, and thus PLEX was discontinued after four sessions (Table 2). Worsening of MDS with symptomatic anemia was the leading diagnosis at this time, and thus, on day 8, erythropoietin 40,000 units weekly was initiated.

**Table 2:** Subsequent laboratory results to guide the differential diagnosis ADAMTS13, a disintegrin and metalloproteinase with thrombospondin type 1 motif, 13

Test	Results	Reference range
ADAMTS13	0.57	0.68-1.63 IU/mL
Serum protein electrophoresis	M-Spike present in the gamma region. Immunofixation shows dual IgM-Lambda monoclonal proteins.	No clonal protein
Quantitative immunoglobins	IgA: 82	66-436 mg/dL
	IgM: 333	43-279 mg/dL
	IgG 814	791-1643 mg/dL
	Total protein: 5.6	5.9-8.3 g/dL
Serum free light chains	Kappa free light chain: 56.6	3.3-19.4 mg/L
	Lambda free light chain: 186.8	5.7-26.3 mg/L
	Kappa/lambda ratio: 0.30	0.26-1.65 ratio
Serum viscosity	1.5	1.5-1.9 relative to H2O
Prostate-specific antigen	51.87 (baseline 1.26 twelve years prior)	≤4 ng/mL
Total testosterone	83 (baseline 244.69 eight years prior)	241-827 ng/dL
Free testosterone	10.9	Not established for age

On days 9-13, the patient was simultaneously managed for other medical problems including acute hypoxic respiratory failure likely secondary to aspiration pneumonia and acute hypoactive hospital-acquired delirium.

**Figure 1:**
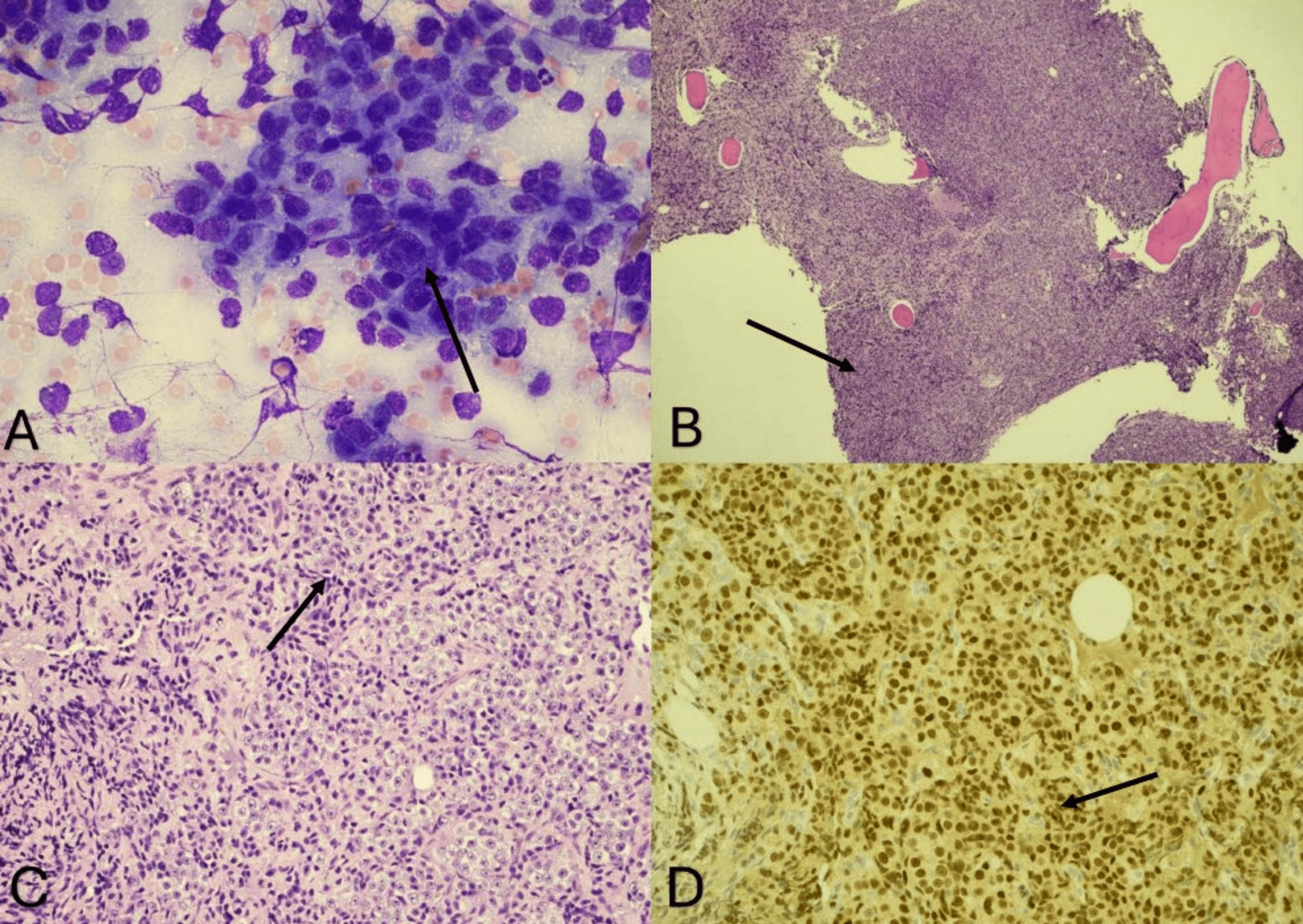
Bone marrow biopsy pathology showing prostatic adenocarcinoma (A) Wright Giemsa aspirate at 400x showing sheets and clusters of metastatic prostatic adenocarcinoma. (B) H&E stain at 40x showing nearly 100% bone marrow replaced by metastatic prostatic adenocarcinoma. (C) H&E stain at 100x with high-power view of infiltrative prostatic adenocarcinoma. The metastatic tumor cells are large vesicular nuclear chromatin and prominent nucleolus, forming sheets, nests, and clusters. (D) NKX3.1 immunostain at 200x shows nuclear positivity, which is consistent with metastatic adenocarcinoma of prostate origin.

On day 14, BM aspirate and core biopsy pathology revealed BM with hematopoietic elements nearly entirely replaced by metastatic adenocarcinoma (Figure 1). Immunostains showed the tumor cells to be positive for NKX3.1 gene and negative for CK7/CK20 or GATA3, findings consistent with metastatic prostate adenocarcinoma and suggestive against bladder cancer. Flow cytometry showed a minute monotypic CD5(dim+)/CD10(-) B-cell population. No secondary lymphoma population was identified. SPEP and serum viscosity were unremarkable, and QIGs showed a mildly elevated IgM (Table 2). Given the diagnosis, PSA and testosterone were measured. PSA was markedly elevated from a baseline 12 years prior, and testosterone level was markedly decreased from a baseline eight years prior (Table 2). It was identified that the patient’s last digital rectal examination (DRE) was around two years prior to presentation at a routine urology appointment, which showed enlarged bilateral lobes without nodularity. Alkaline phosphatase (ALP) was normal on admission. The patient was informed of these findings and consented to begin treatment with bicalutamide 50mg daily for 14 days for short-term androgen blockade to reduce tumor flare (of which four doses were administered inpatient), followed by androgen deprivation therapy (leuprolide 22.5mg) every three months. A prostate-specific membrane antigen positron emission tomography combined with computed tomography (PSMA-PET/CT) for staging was planned for the outpatient setting.

After 18 days of hospitalization, the patient was discharged to a skilled nursing facility with resolution of his other medical problems, and outpatient follow-up was planned. After initially missing his first leuprolide injection scheduled for eight days post-discharge, he eventually arrived at a rescheduled appointment a week later and received his initial leuprolide injection. Subsequent attempts at follow-up were unsuccessful, and thus the patient missed his PSMA-PET/CT scheduled for three weeks post-discharge and missed three attempted oncology clinic appointments initially scheduled for six weeks post-discharge. The patient was eventually found to be deceased at home during a wellness check five months after the initial presentation.

## Discussion

We presented the case of a 91-year-old man who presented with clinical signs of TMA and was managed empirically for TTP with daily PLEX and high-dose steroids. He was later found to have normal ADAMTS13 activity; in the interim, the patient underwent a BM biopsy with findings of prostate adenocarcinoma, which ultimately explained his presentation. TMA is a clinicopathological diagnosis of thrombocytopenia, anemia, and RBC fragmentation (e.g., schistocytes), with primary causes including TTP and hemolytic uremic syndrome. However, this patient likely developed TMA secondary to urosepsis and underlying malignancy. Still, high early mortality in TTP (around 50% of deaths occur in the first 24 hours) necessitates empiric treatment when thrombocytopenia and schistocytic anemia are present, as was appropriately done in this case. A “PLASMIC” score of ≥5 can help guide the need for further work-up.

**Figure 2:**
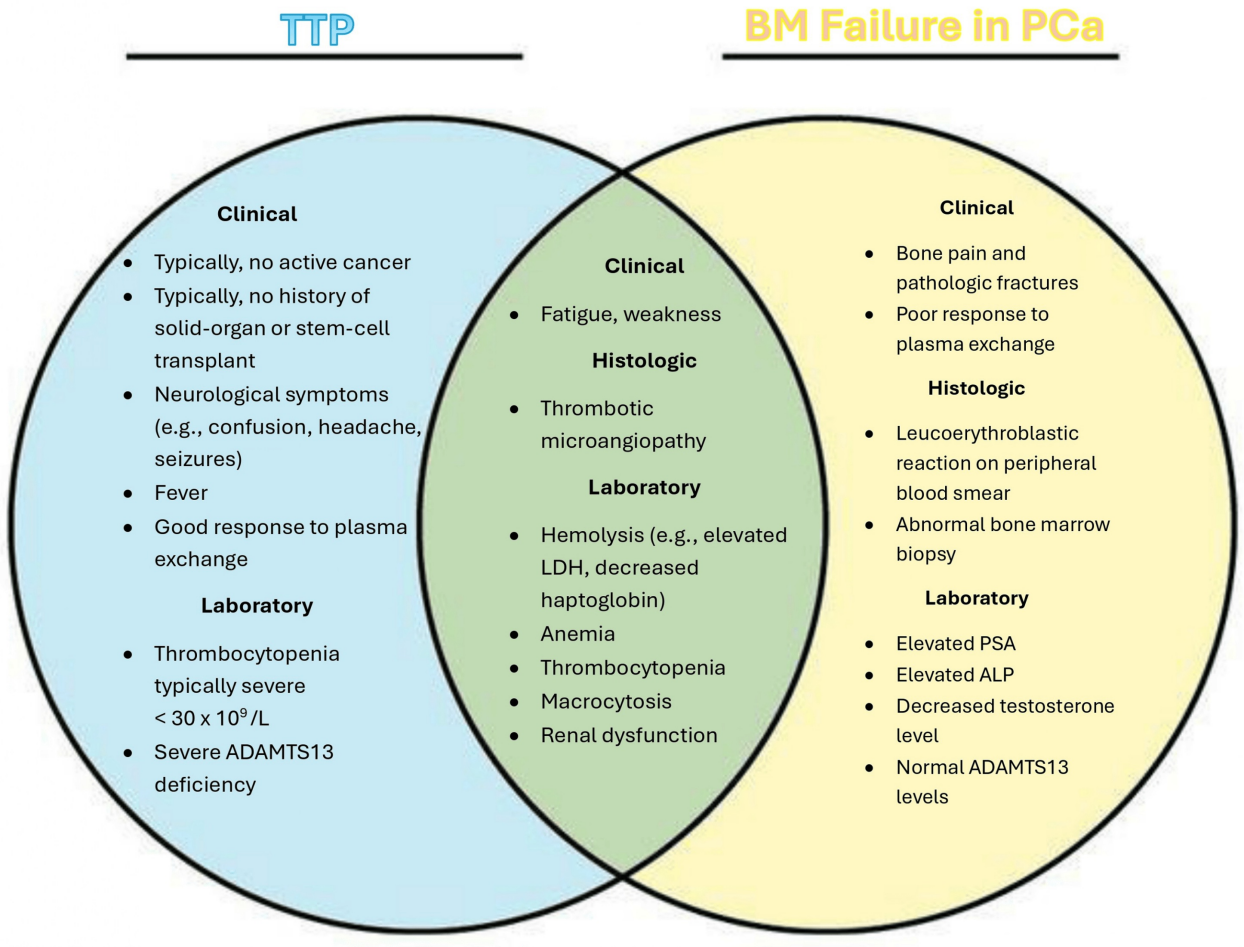
Venn diagram comparing typical features of TTP versus BM failure in PCa ADAMTS13, a disintegrin and metalloproteinase with thrombospondin type 1 motif 13; ALP, Alkaline phosphatase; BM, bone marrow; PCa, Prostate cancer; PSA, prostate-specific antigen; TTP, thrombocytopenic purpura

Features that further complicated this case included the history of IgM MGUS and MDS, which added complexity to diagnostic considerations, and the presence of a chronic indwelling Foley due to known BPH may have masked the progression of worsening lower urinary tract symptoms in the setting of his cancer. Furthermore, despite the history of known BPH, the patient lacked routine PSA monitoring. As mentioned, the patient’s last DRE was around two years prior to presentation at a routine urology appointment, which showed enlarged bilateral lobes without nodularity, but more frequent examinations may have been warranted. PSA screening does not result in PCa-specific or overall survival benefit, but current clinical practice guidelines use the clinical symptoms, risk factors (older age, African descent, family history), DRE, and shared decision-making to guide concern for further screening or diagnostic measures. While PSMA-PET/CT was never performed, it is curious whether the patient’s chronic rib fractures are the sequalae of bone lytic metastatic lesions. The features of TTP are compared and contrasted with the features of BM metastasis in PCa in Figure 2.

One case report describes pancytopenia as an initial manifestation of PCa without urological or bone-related symptoms and normal prostate, ultimately without response to degarelix and managed palliatively [9]. Another case report similarly describes a patient presenting with dyspnea, nausea, vomiting, and pancytopenia found to have BM, lung, duodenal, and colonic metastases [10]. Other unusual metastatic sites at initial presentation of prostate adenocarcinoma have been described [11].

The most common sequela of PCa metastasis is bone osteoblastic lesions. Metastasis of PCa to bone resulting in osteoblastic and/or osteolytic lesions is well characterized. PCa cells cause osteoblastic metastasis through release of osteoblast-promoting factors such as bone morphogenic protein, Wnt family ligand, endothelin-1, platelet-derived growth factor, and osteolytic lesions through the release of parathyroid hormone-related protein (PTHrP), which upregulate receptor activator of nuclear factor kappa-B ligand (RANKL) and down-regulate osteoprotegerin to result in osteoclastogenesis and bone resorption [12-13]. Bone metastasis can cause intense bone pain, pathological fractures, and hypercalcemia. Patients with higher PSA and ALP levels tend to show more bone lesions, but, when high PSA is present, PSA response to androgen deprivation therapy is a reliable predictor of survival [14]. Median survival of patients with PCa who have bone metastasis is less than three years, with an average five-year survival rate of 3% [15].

The pathogenesis of BM involvement in PCa has been explored as well. PCa cells express C-X-C chemokine receptor type 4 (CXCR4), which causes their homing to BM by C-X-C motif chemokine 12 (CXCL12)/CXCR4 signaling to compete with hematopoietic stem cells; further involvement by activation of the phosphatidylinositol 3-kinase (PI3K) and protein kinase B (Akt) pathway and higher presence of regulatory T cells in BM metastatic PCa lead to higher levels of CXCR4 to further drive spread [13]. Osteonectin, sonic hedgehog (Shh), and other factors all play a role in BM metastatic PCa as well [13]. Further understanding of the pathogenesis of BM involvement has led to various therapeutic considerations, especially considering that antitumor therapy could improve overall survival in patients with BM metastasis [16]. One study identified that myeloid-like tumor hybrid cells promote progression of bone metastasis and could serve as a potential therapeutic target [17]. Akt inhibitors may potentially be used to inactivate CXCL12/CXCR4 signaling to target BM metastasis in PCa as well [18].

The clinical significance and oncological prognosis of BM metastasis in PCa remain largely unclear though briefly explored. One retrospective review of 103 bone metastatic castrate-resistant PCa cases reported that those with ≤10 bone lesions had a significantly longer time to develop cytopenias, including severe cytopenia events, than those with >10 lesions, suggesting that the extent of bone metastases may predict BM involvement and resulting cytopenias [19]. A non-significant trend to poorer progression-free survival and overall survival in patients with BM involvement was noted in correlation with poor functional status, higher ALP, and lower Hgb [8].

## Conclusions

This case highlights the diagnostic complexity of cytopenias. While TTP-like features were immediately evident, the patient’s TMA could be attributed to malignancy and infection, and, ultimately, his cytopenias were due to BM failure from mCSPC. The patient’s MDS further complicated the differential. Despite the diagnostic challenge, empiric TTP treatment is critical given the high 24-hour mortality, with “PLASMIC” scoring assisting decision-making. In refractory cases, a high index of suspicion should be retained for BM infiltrative processes. The case also underscores the importance of PCa screening; this patient lacked routine PSA monitoring despite known BPH, and chronic Foley catheter use may have masked worsening lower urinary tract symptoms. Finally, the prognostic implications of BM metastasis in mCSPC warrant further research. Existing studies suggest that high bone metastatic burden (likely contributing to the patient’s chronic fractures) may predict BM involvement and resulting cytopenias, with trends to poorer survival. Future studies should explore therapeutic avenues targeting the pathophysiology of BM metastasis, such as using Akt inhibitors in clinical practice or targeting myeloid-like tumor hybrid cells.
